# Comparative morphology of ultimate and walking legs in the centipede *Lithobius forficatus* (Myriapoda) with functional implications

**DOI:** 10.1186/s40851-018-0115-x

**Published:** 2019-01-14

**Authors:** Matthes Kenning, Vanessa Schendel, Carsten H. G. Müller, Andy Sombke

**Affiliations:** 1 Cytology and Evolutionary Biology, University of Greifswald, Zoological Institute and Museum, Soldmannstrasse 23, 17489 Greifswald, Germany; 2 General and Systematic Zoology, University of Greifswald, Zoological Institute and Museum, Loitzer Strasse 26, 17489 Greifswald, Germany; 3 Centre for Advanced Imaging, The University of Queensland, Building 57, St. Lucia, Queensland 4072 Australia; 4 Department of Integrative Zoology, University of Vienna, Althanstrasse 14, 1090 Vienna, Austria

**Keywords:** Chilopoda, Evolution, microCT, Neuroanatomy, Nervous system, Scanning electron microscopy, Backfilling

## Abstract

**Background:**

In the context of evolutionary arthopodial transformations, centipede ultimate legs exhibit a plethora of morphological modifications and behavioral adaptations. Many species possess significantly elongated, thickened, or pincer-like ultimate legs. They are frequently sexually dimorphic, indicating a role in courtship and mating. In addition, glandular pores occur more commonly on ultimate legs than on walking legs, indicating a role in secretion, chemical communication, or predator avoidance. In this framework, this study characterizes the evolutionarily transformed ultimate legs in *Lithobius forficatus* in comparison with regular walking legs.

**Results:**

A comparative analysis using macro-photography, SEM, μCT, autofluorescence, backfilling, and 3D-reconstruction illustrates that ultimate legs largely resemble walking legs, but also feature a series of distinctions. Substantial differences are found with regard to aspects of the configuration of specific podomeres, musculature, abundance of epidermal glands, typology and distribution of epidermal sensilla, and architecture of associated nervous system structures.

**Conclusion:**

In consideration of morphological and behavioral characteristics, ultimate legs in *L. forficatus* primarily serve a defensive, but also a sensory function. Moreover, morphologically coherent characteristics in the organization of the ultimate leg versus the antenna-associated neuromere point to constructional constraints in the evolution of primary processing neuropils.

## Background

The last pair of legs (ultimate legs) of centipedes are evidently exceptional, as no other centipede legs are of comparable functional, morphological, and behavioral heterogeneity [1]. Although the venomous forcipules are a textbook example of arthropodial transformations and a hallmark of centipedes, they always hold a common functional coherence in prey capture and killing. A plethora of behavioral adaptations is associated with the evolutionary transformations of the ultimate legs. They are rarely used for locomotion, if at all. In most adult centipedes they are the largest legs and recognizable by their shape and aspect, and by the way they are moved in comparison to regular walking legs. Many species possess significantly elongated, thickened, or pincer-like ultimate legs, and frequent sexual dimorphisms indicate a pivotal role in courtship and mating. In addition, glandular pores are much more often present on ultimate legs than on walking legs, signifying their relevance in secretion, chemical communication, or predator avoidance (summarized by [1]). Furthermore, the morphology of the ultimate legs and their podomeres, as well as the occurrence of sensilla and epidermal glands not only holds a high taxonomical value, they also are a promising example for studying and tracing functional and constructional aspects of leg modifications.

**Fig. 1:**
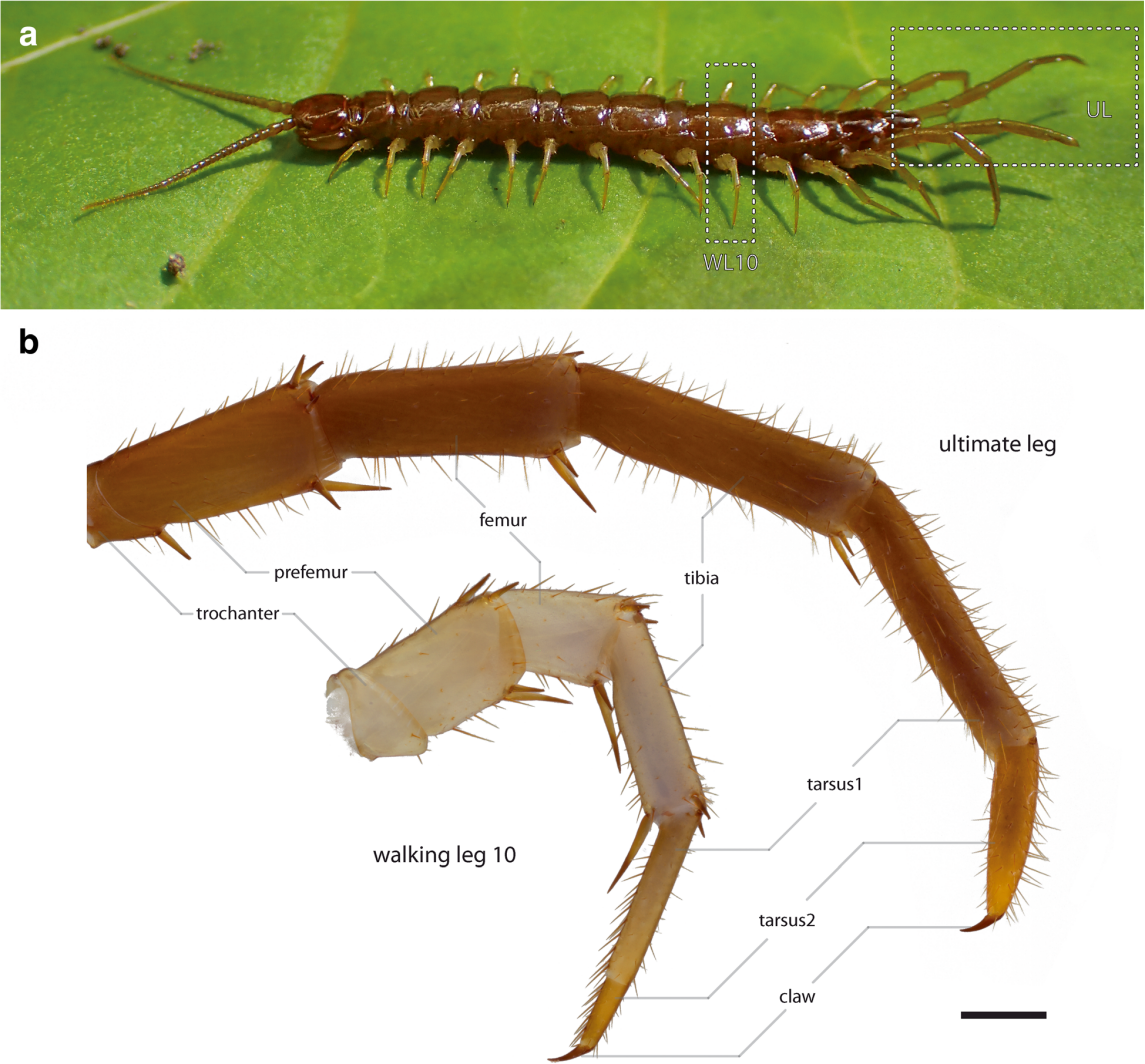
*Lithobius forficatus*. **a** Habitus, view from lateral. Dashed rectangles indicate walking legs 10 and the ultimate legs. Note that the left ultimate leg (front) was damaged and regenerated. **b** Macrophotographs of ultimate leg (top) and walking leg 10 (bottom) from the same specimen, to scale. Coxae of both legs not shown, trochanter only partially shown. Note the difference in length of corresponding podomeres. Scale bar 500 μm

Lithobiomorph centipedes possess 17 post-cephalic pairs of trunk appendages: the forcipules, 14 walking legs, the ultimate legs (Fig. 1), and the gonopods. The ultimate legs are in most cases also easily recognized by their size in comparison to walking legs [2–4], and how they are held parallel to each other and lifted slightly above the ground, or occasionally dragged behind the animal. The last four pairs of legs display a gradual increase in length and possess a presumed defensive function, although this is mainly mediated by the ultimate legs alone [5–8]. When threatened, many lithobiids lift the ultimate legs in a defensive display, followed by twitching up- and downward movements, and the secretion of a sticky substance [8–10]. This secretion becomes effective in avoiding predation. The release sites of this secretion are closely aggregated pores of so-called telopodal glands, situated on the medial faces of the distal podomeres (i.e., femur, tibia, and tarsi) [11]. These telopodal glands are common in various taxa of Lithobiidae, whereas among Henicopidae they have only been documented in *Lamyctes fulvicornis* [12, 13]. Histochemistry has revealed that the sticky fibers produced by telopodal glands are made of a lipoid-protein complex resembling silk [12]). With respect to general anatomy and morphology, and other biological fields, such as endocrinology, development, and physiology, the centipede *Lithobius forficatus* (Linnaeus, 1758) is one of the best studied myriapod species [14]. Nevertheless, in the field of sensory biology, much remains unknown. In fact, only a few physiological and behavioral studies have been conducted so far [15–18], demonstrating responses to various chemical, visual, and physical stimuli (e.g. thermo-, hygro-, mechanoreception). In addition, antennal sensilla and the associated processing structures in the brain were investigated in greater detail [5, 19–22]. However, we have only begun to explore the complexity of the architecture of the peripheral and central nervous systems, especially of post-cephalic appendages that display a high morphological and functional variability (forcipules, walking legs, and ultimate legs). Besides the brain, the ventral nerve cord of *L. forficatus* is composed of mostly well-separated ganglia: the subesophageal ganglion, the forcipular ganglion, 15 leg-associated ganglia, and the terminal (or postpedal) ganglion [23, 24]. Within ganglia of the arthropod ventral nerve cord, different sensory modalities often segregate to distinct synapse-dense regions (summarized by [25]). For example, in hexapod thoracic ganglia, afferents from mechanoreceptive sensilla project to three distinct regions [26]. Concerning a given sensory modality, an ordered structure of neuropilar areas is usually observed along a gradient (i.e. odotopy and/or somatotopy) [25], shaping the entire neuronal substrate accordingly. In *L. forficatus’* walking leg ganglia, afferents from the leg-associated nerves 4 and 5 innervate a small, so-called ventral neuropilar domain that is thought to be the primary processing center integrating sensory information [24].

The question thus arises whether the pronounced length of ultimate legs is correlated with a higher abundance of cuticular sensilla whose axons, as a general consequence, shape primary processing neuropils in the ultimate leg associated ganglion, as well. This plasticity cascade is mostly known and well documented for the mandibulate deutocerebrum that is characterized by the bipartite nature of how sensory information is processed. Innervated by axons of a variety of antennal sensilla comprising different modalities, it is typically composed of a neuropil responsible for processing chemosensory information (i.e. deutocerebral chemosensory lobe) and at least one mechanosensory neuropil (i.e. corpus lamellosum in Myriapoda, AMMC in Hexapoda, lateral antenna 1 neuropil in Crustacea) [22, 25, 27–32].

Along these lines, this study sets out to characterize the evolutionarily transformed ultimate legs (sensu Kenning et al. [1]) in *Lithobius forficatus* in comparison with the “regular” walking leg 10. This contribution aims at gaining insights on qualitative disparities and functional implications in terms of general morphology, musculature, typology and distribution of epidermal sensilla, and the architecture of the associated nervous system.

## Materials and methods

### Experimental animals

Adult individuals of *Lithobius forficatus* (Linnaeus, 1758) were collected under dead wood in and around Greifswald (Germany) and kept individually in plastic boxes or together in a terrarium. They were provided with water once a week and fed small crickets (*Acheta domesticus*) every 2 weeks. Species determination was done according to Barber [3].

### Macro-photography

For macro-images, one specimen was fixed in 70% ethanol and documented using the BK PLUS Lab system (Dun Inc., http://www.duninc.com/bk-plus-lab-system.html) with a customized Canon MPE 65 mm 1–5x micro-photography lens mounted on a Canon 6D camera. Image stacks were captured with Adobe Lightroom and processed using Zerene Stacker under PMax value.

### Scanning electron microscopy

After anesthetization using carbon dioxide (dry ice), four male and five female adult specimens were fixed in 70% ethanol for 24 h, transferred to glass vials and cleaned in ethanol using an ultrasonic bath. After dissection and dehydration in a graded ethanol series (70 to 99%), specimens were critical-point-dried using the automated dryer, Leica EM CPD300 (Leica Microsystems), and mounted on copper wire (PLANO #16067) or carbon-conducted tabs (PLANO #G3347). Samples were gold-palladium sputter-coated and examined with a Zeiss EVO LS10 at 10 kV at the Imaging Center of the University of Greifswald.

### X-ray micro-computed tomography (microCT)

After anesthetization, two adult specimens were fixed in Bouin’s solution overnight. The subsequent preparation followed the protocol by Sombke et al. [33]. Preparations were rinsed in several changes of PBS (phosphate buffered saline, 0.1 M, pH 7.4), dehydrated in a graded ethanol series and incubated in a 1% iodine solution (iodine resublimated in 99% ethanol; Carl Roth #X864.1) for 12 h. Preparations were rinsed several times in pure ethanol and critical-point-dried. Finally, samples were fixed on insect pins with super glue. Scans were performed with a Zeiss Xradia MicroXCT-200. Dissected walking legs (pair 10) were scanned with a 10× objective lens resulting in a 2.19 μm pixel size; ultimate legs were scanned with a 4× objective lens unit resulting in 5.05 μm pixel size. Dissected ventral nerve cord ganglia associated with walking legs were scanned with a 20x objective lens resulting in a 0.93 μm pixel size (compare also Schendel et al. [24]). The ultimate leg associated ganglion 15 was scanned using a 20× objective lens resulting in a 1.09 μm pixel size. Tomography projections were reconstructed using the XMReconstructor software (Zeiss Microscopy) resulting in image stacks (TIFF format). All scans were performed using binning 2 (resulting in noise reduction) and subsequently reconstructed using binning 1 (full resolution) to avoid information loss.

### Autofluorescence preparation

For autofluorescence contrast enhancement, two specimens were anesthetized and fixed in a mixture of 4% paraformaldehyde and 4% glutaraldehyde (1:1) at 4 °C for 2 days [22]. After several washing steps in PBS, ventral nerve cords were dissected, dehydrated in a graded series of ethanol, and embedded in methyl salicylate (Sigma Aldrich #W274518). Preparations were analyzed using a Leica SP5 II confocal laser scanning microscope. A double excitation of 488 nm and 561 nm was used to detect and visualize autofluorescence of the nervous tissue.

### Backfills of leg nerves

For backfill experiments, four specimens were anesthetized, wrapped in fine gauze in order to restrict movements but enable air circulation, and pinned on a piece of cardboard. While still anesthetized, ultimate legs were cut at the basal prefemur and pieces of the cuticle were removed in order to expose the leg nerve. A short preincubation with a capillary filled with distilled water was followed by injection with a solution of 3% Lucifer yellow (ThermoFisher #L453) in distilled water for 8–24 h at 4 °C using a capillary placed over the remaining leg. Fixation in 4% paraformaldehyde was followed by dissection and several washing steps in PBS, dehydration in a graded series of ethanol, and clearing in methyl salicylate. All preparations were examined using a Leica SPII confocal laser scanning microscope.

### Image processing and terminology

Segmentation and volume rendering of image stacks based on microCT and cLSM analyses were performed using AMIRA 6.0.1 (FEI). Based on backfills, 3D models of the termination sites were prepared and morphometric parameters such as volume were analyzed. Images were processed in Adobe Photoshop using global contrast and brightness adjustment features as well as black and white inversion. Depth-coded projections were generated using Fiji (ImageJ v. 1.51f). The neuroanatomical nomenclature is according to Loesel et al. [25] and Schendel et al. [24], whilst the numbering of muscles and nerves is according to Rilling [34].

## Results

### External morphology of walking leg 10 and ultimate leg

**Fig. 2:**
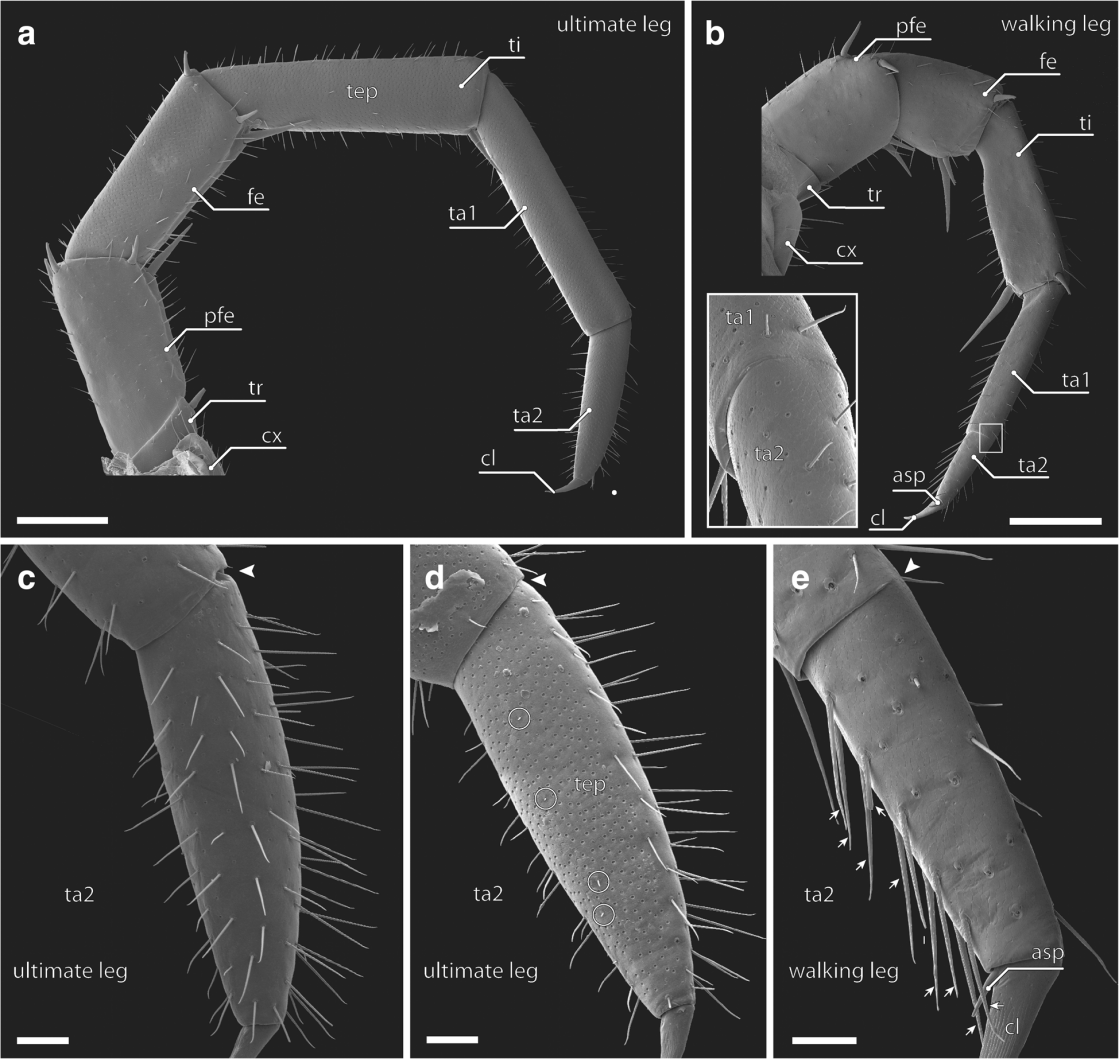
External morphology of walking and ultimate leg. **a** Overview of ultimate leg, from medial. Scale bar 500 μm. **b** Overview of walking leg 10, from medial. Note that only the walking leg features an accessory spine on the claw (asp, compare **e**). Inset shows the dorsally incomplete tarsal articulation of walking legs (compare **d**–**e**). Scale bar 500 μm. **c** Ultimate leg tarsus 2 from lateral. The arrowhead indicates the complete tarsal articulation. Scale bar 100 μm. **d** Ultimate leg tarsus 2 from medial. Pores of the telopodal organs (tep) cover the entire medial surface. Note the four sensilla mesotrichodea II interspersed between the telopodal gland pores (circles). The arrowhead indicates the complete tarsal articulation (compare **c**, **d**). Scale bar 100 μm. **e** Tarsi of walking leg 10, from medial. The arrowhead indicates the incomplete articulation of the tarsi. Arrows point to s. macrotrichodea II that are only present on the ventral side of walking leg tarsus 1 (compare Figs. 5 and 6h). Scale bar 50 μm. Abbreviations: asp accessory spine, cl claw, cx coxa, fe femur, pfe prefemur, ta tarsus, tep telopodal gland pores, ti tibia, tr trochanter

In *Lithobius forficatus*, ultimate legs are distinctly longer than walking leg 10 (Fig. 1) and of identical podomere configuration in both sexes. However, in walking legs the tarsi are fused at the dorsal side, while in ultimate legs they are clearly separated (Fig. 2c–e, arrowhead). In addition, the claw on walking legs 1–14 possesses an accessory spine that is absent from the ultimate leg claw (Fig. 2d, e).

### Pores and epidermal glands

**Fig. 3:**
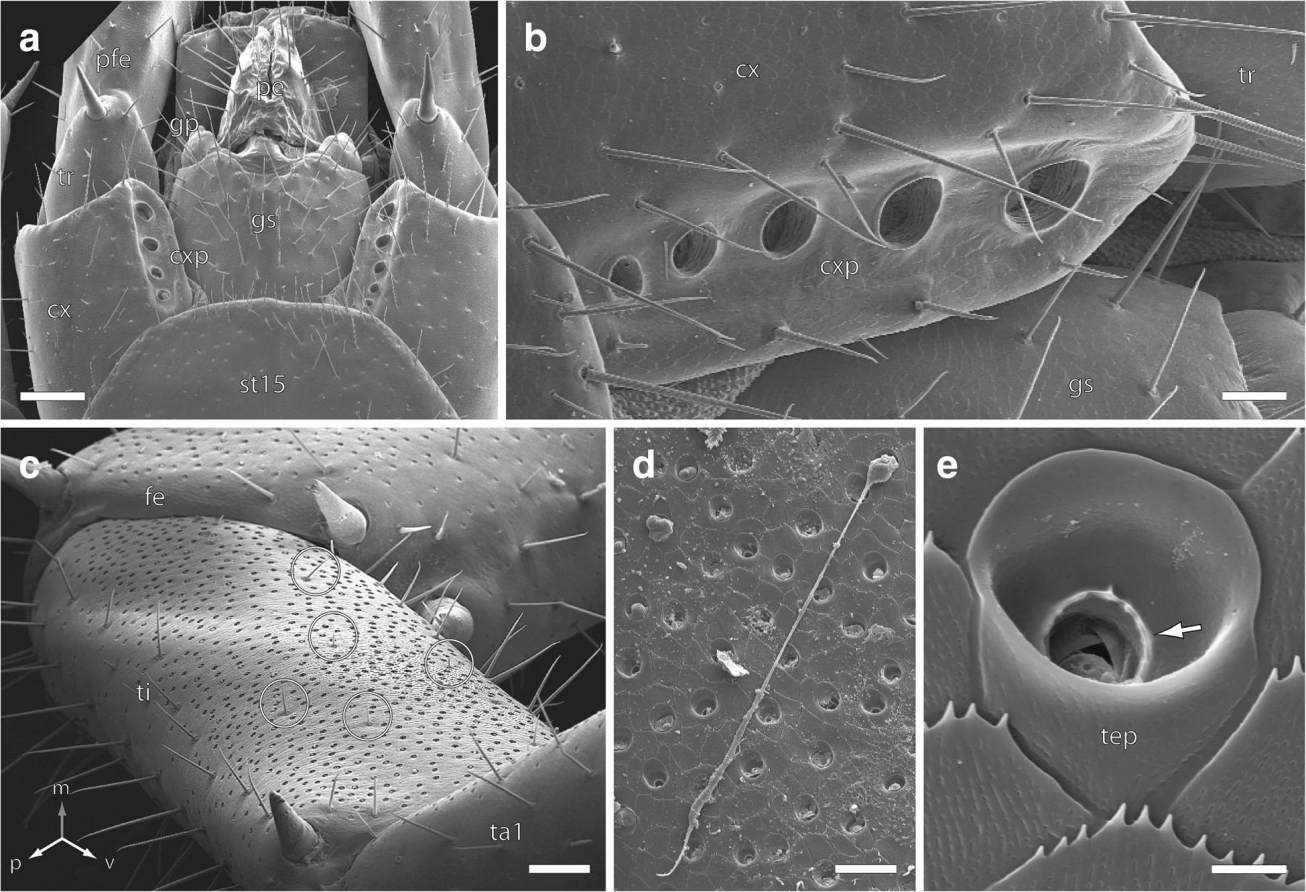
Terminal trunk region of *Lithobius forficatus*. **a** Ventral view of male terminal trunk, ultimate leg coxae with coxal pores, and gonopods. Scale bar 200 μm. **b** Close up on left ultimate leg coxa with coxal pores, ventral view. Scale bar 40 μm. **c** Medial face of ultimate leg femur, tibia, and tarsus 1. Note the higher abundance of telopodal gland pores on the tibia. Except for s. mesotrichodea II (circles), the medial faces are devoid of sensilla. Scale bar 300 μm. **d** Closely aggregated pores of telopodal glands on the tibia with secretion. Scale bar 20 μm. **e** Detail of a telopodal gland pore. Note the dentate collar at the bottom of the pit, through which the glandular duct opens (arrow). Scale bar 3 μm. Abbreviations: cx coxa, cxp coxal pores, fe femur, gp gonopods, gs sternite of gonopodial segment, pe penis, st sternite, ta tarsus, tep telopodal gland pores, ti tibia, tr trochanter

The last four pairs of legs (12–15) have a special status in terms of their association with large pores and numerous epidermal exocrine glands. The ventral face of the coxae of these legs feature 5–7 large elliptical pores (diameter ~ 50–60 μm, depth ~ 40 μm), known as coxal pores (Fig. 3a, b). In addition, the medial faces of the last four podomeres (femur, tibia, tarsus 1, and tarsus 2) of leg pairs 12–15 are covered with pores of the telopodal glands; shallow, but complex openings in the cuticle measuring about 5 μm in diameter (Figs. 2a, d; 3c–e). Each pore is surrounded by a small elliptical cuticular plate with a shallow pit at the base of which a dentate collar encloses the glandular duct (Fig. 3d, e). While there are roughly 250 pores on leg 12, 1000 pores on leg 13, and 3300 on leg 14, each ultimate leg harbors about 4000 pores (*n* = 2), showing a huge variation between the single podomeres (Figs. 2a, d; 3c). Although the total number decreases distally due to the decreasing surface area of each podomere (femur: 855, tibia: 1380, tarsus 1: 1130, and tarsus 2: 481), the density continuously increases from about 20 pores per 10,000 μm^2^ (i.e. 100 × 100 μm) on the femur to 32 pores on tarsus 2 (Fig. 3c).

### Anatomy of walking leg 10 and ultimate leg - musculature and tendons

**Fig. 4:**
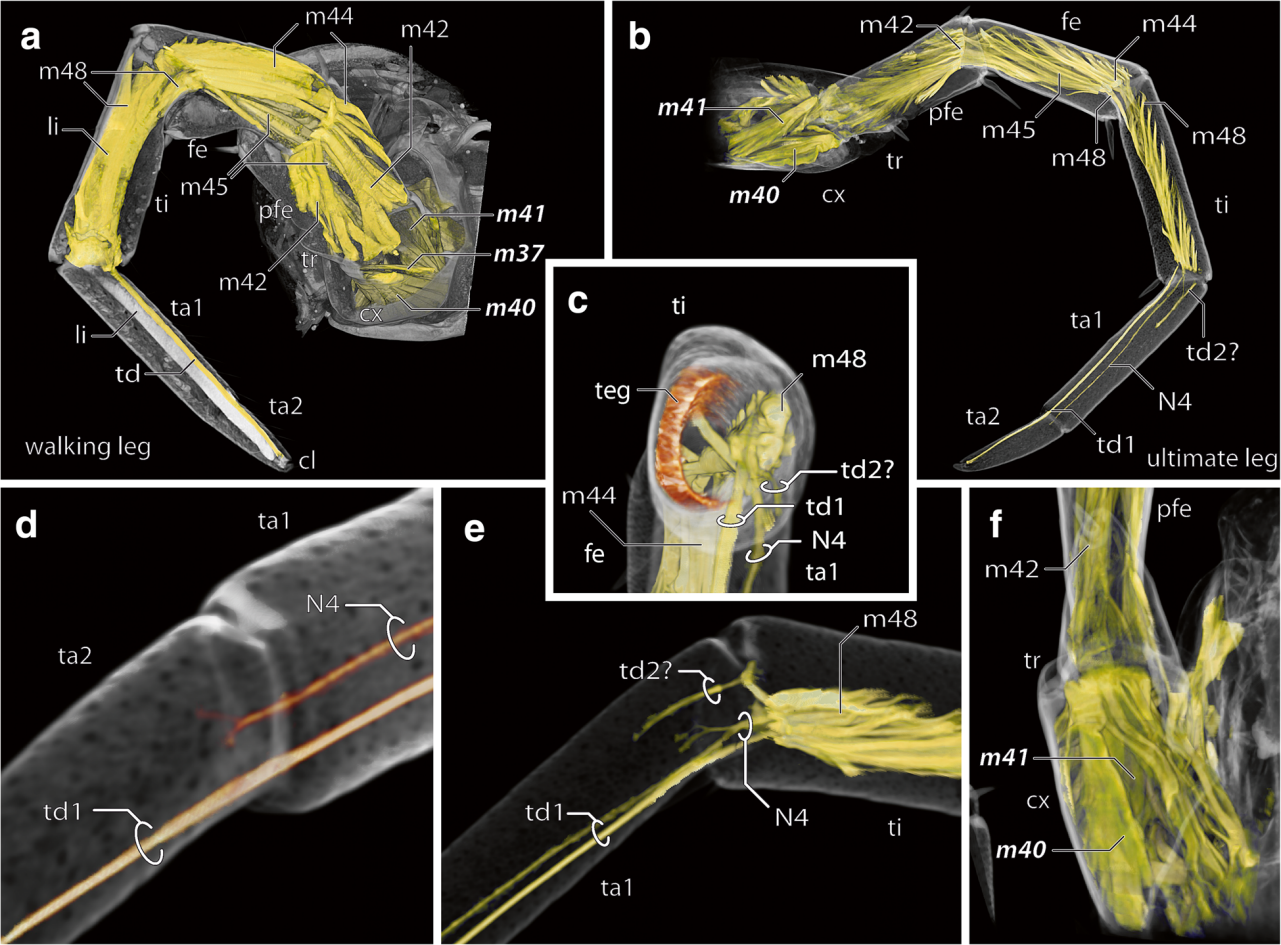
Anatomy of walking leg 10 and ultimate leg. **a** 3D volume rendering of walking leg 10 and musculature, microCT analysis. Note that muscles labeled in bold either exhibit differences to ultimate leg muscles (m40 and m41, compare **b**) or are absent in the ultimate leg (m37). **b** 3D volume rendering of ultimate leg and musculature, microCT analysis. Note that muscles labeled in bold exhibit differences to walking leg 10 (compare **a**). **c** Detail of ultimate leg tibia showing ventrolaterad displacement of the claw tendon (td1), nerve N4, and muscle m48 by the tissue of the telopodal glands (teg). **d** Ultimate leg articulation of tarsus 1 and 2 with N4 branching in the proximal part of tarsus 2. Pores of telopodal glands are detectable in this visualization. **e** Tibio-tarsal articulation of the ultimate leg showing the branching of N4 in the proximal tarsus 1. Tarsus 1 houses a presumptive second tendon (td2?) supplied by a single muscle head from m48. **f** Right ultimate leg coxa from ventral. Note that the levator and depressor muscles m40 and m41 are substantially enlarged (compare **a**). Abbreviations: cl claw, cx coxa, fe femur, li connective tissue ligament, m muscle. N nerve, pfe prefemur, ta tarsus, td1 claw tendon, td tendon, teg telopodal gland tissue, ti tibia, tr trochanter

The composition of intrinsic musculature is essentially identical in the telopodite of walking and ultimate legs (Fig. 4a, b). However, due to the difference in size, they appear prolate and rather unwoven in the ultimate leg (Fig. 4b); this is particularly the case for flexors in the femur (m42) and tibia (m44). Moreover, muscle head attachment sites, leg nerve N4, and claw tendons are displaced ventrolaterally by the glandular tissue of the telopodal glands in the femur and tibia (Fig. 4c). In both legs, a large tendon connects the claw with the large musculus flexor praetarsi (m48) that proceeds distally alongside N4 (Fig. 4a–e). In the ultimate leg, the leg nerve N4 branches at two positions: at the tibio-tarsal articulation within tarsus 1, giving rise to a small arborization; and again at the tarsal articulation within tarsus 2, after which the nerve could not be traced further (Fig. 4d, e).

In walking and ultimate legs, the single heads of m48 are attached to the tibia and the femur. In the walking leg, however, several additional muscle heads are also connected to prefemur and trochanter. Whether this is also the case in the ultimate leg is inconclusive. The claw tendon in the walking leg is connected to the claw, and also to the tarsi by means of a connective tissue ligament, apparently absent in the ultimate leg (Fig. 4a, b). There is, however, a presumptive second tendon in the ultimate leg at the tibio-tarsal articulation that is supported by a small muscle head of m48 (Fig. 4b, c, e). The tendon could only be traced for a couple of mm, after which it disintegrates (at least in our preparations) within the telopodal gland tissue (Fig. 4e). The most prominent difference of walking and ultimate legs, however, concerns muscle patterns within the coxa (Fig. 4f). In contrast to walking legs 1–14 (compare Fig. 4a), there is no trace of the sterno-trochanteral promotor (m37) in the ultimate leg, and the coxo-trochanteral levators and depressors (m40 and m41) are substantially enlarged.

### Sensilla of walking leg 10 and ultimate leg

**Fig. 5:**
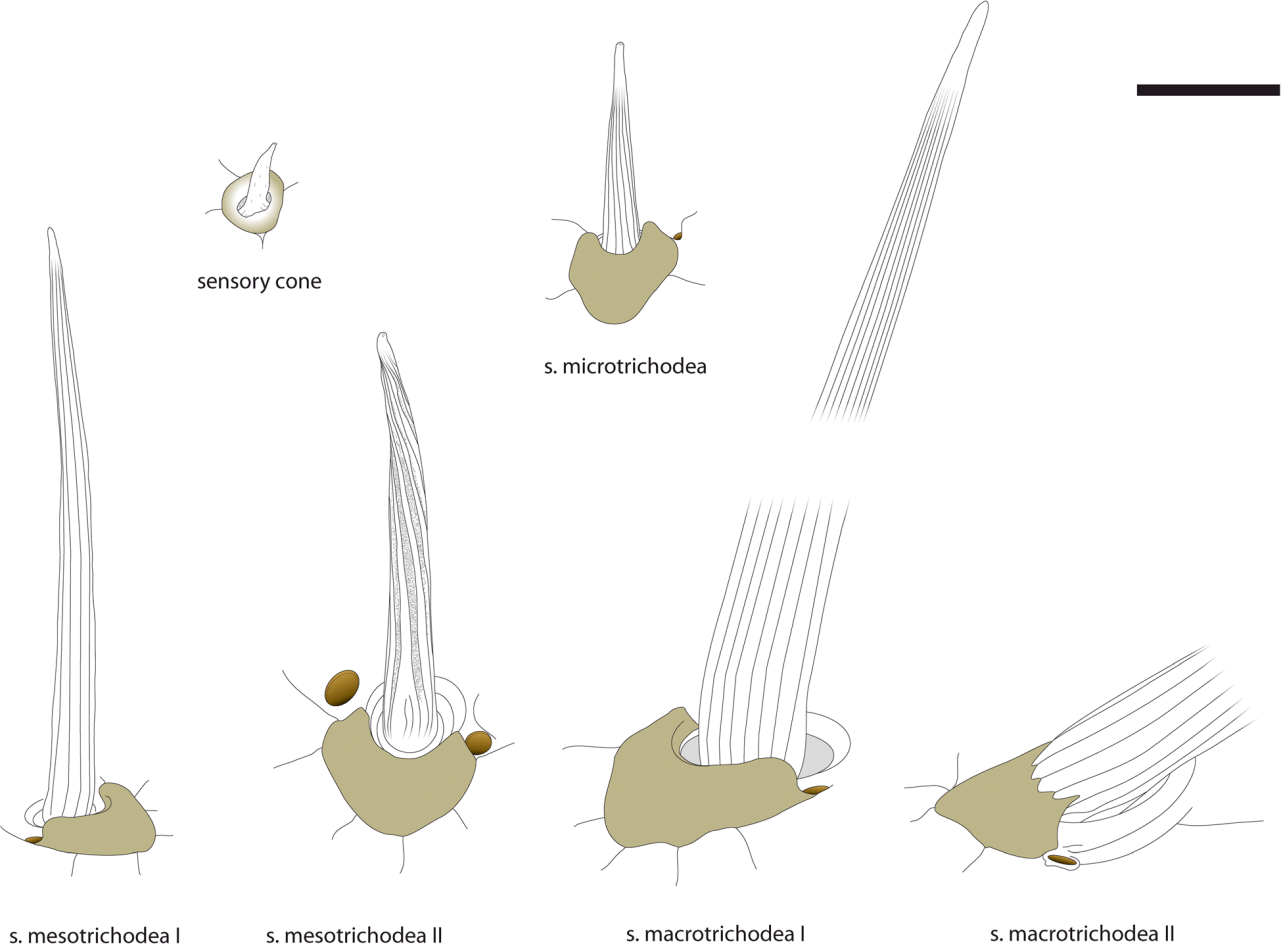
Compilation of schematic drawings of sensilla types found on walking leg 10 and ultimate leg. Scale bar 10 μm

**Fig. 6:**
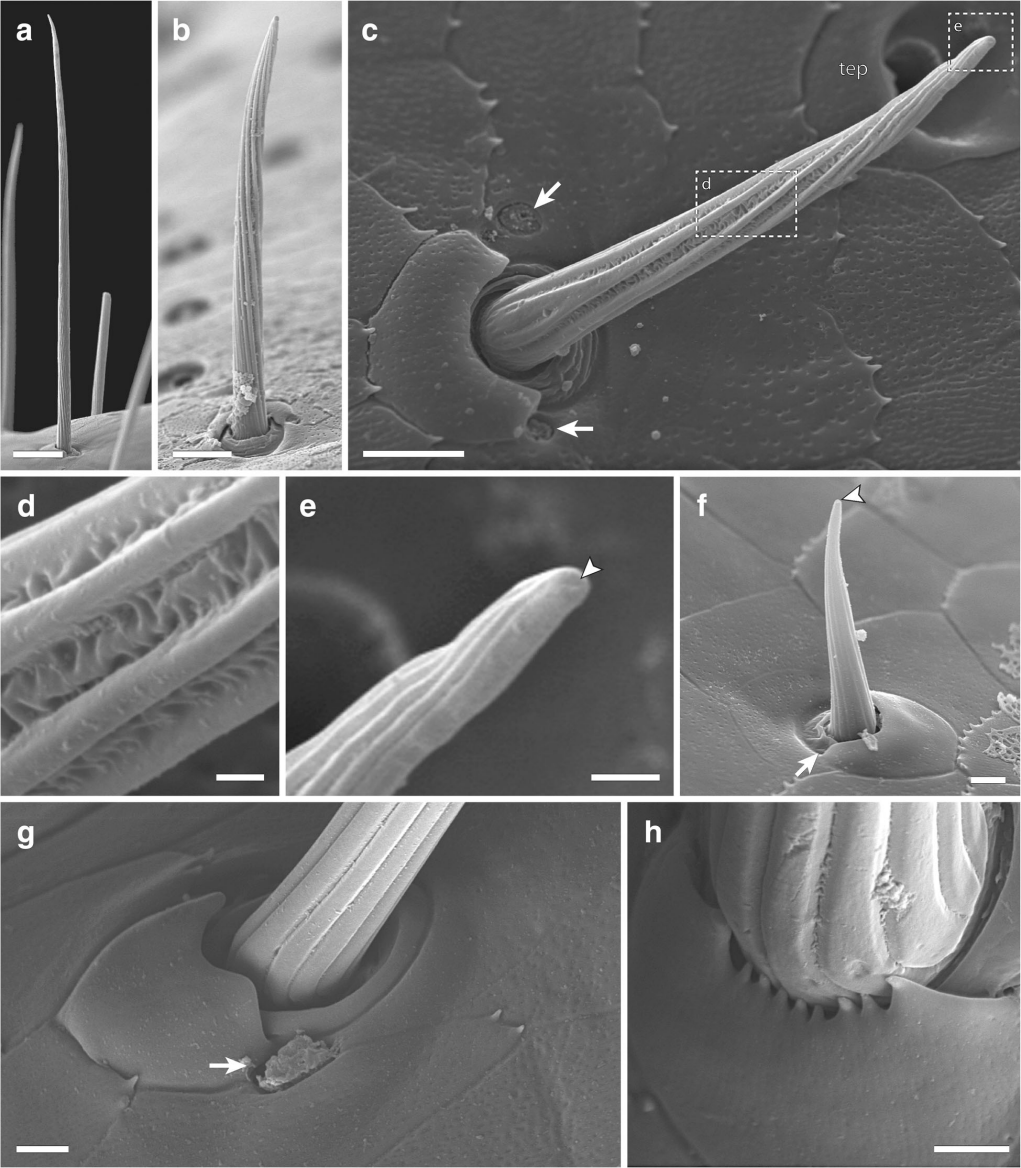
Sensilla types on walking and ultimate legs. **a** Sensillum macrotrichodeum I. Note the orientation of the sensillum’s shaft nearly perpendicular to the cuticle. Scale bar 10 μm. **b** Sensillum mesotrichodeum I. Scale bar 10 μm. **c** Sensillum mesotrichodeum II. Note the association with two pores of solitary epidermal glands (arrows), and the characteristic appearance of the shaft. Scale bar 5 μm. **d** Detail on the sensillar shaft as indicated in **c** illustrating the sponge-like cuticle between the ribs. Scale bar 500 nm. **e** Apex of s. mesotrichodeum II featuring a small terminal pore (arrowhead). Scale bar 1 μm. **f** Sensillum microtrichodeum with terminal pore. Scale bar 2 μm. **g** Crescent shaped socket of s. macrotrichodeum I. The sensillum is accompanied by a single glandular pore. Scale bar 2 μm. **h** Close up on multidentate socket of s. macrotrichodeum II. Scale bar 2 μm. Abbreviations: tep telopodal gland pore

**Fig. 7:**
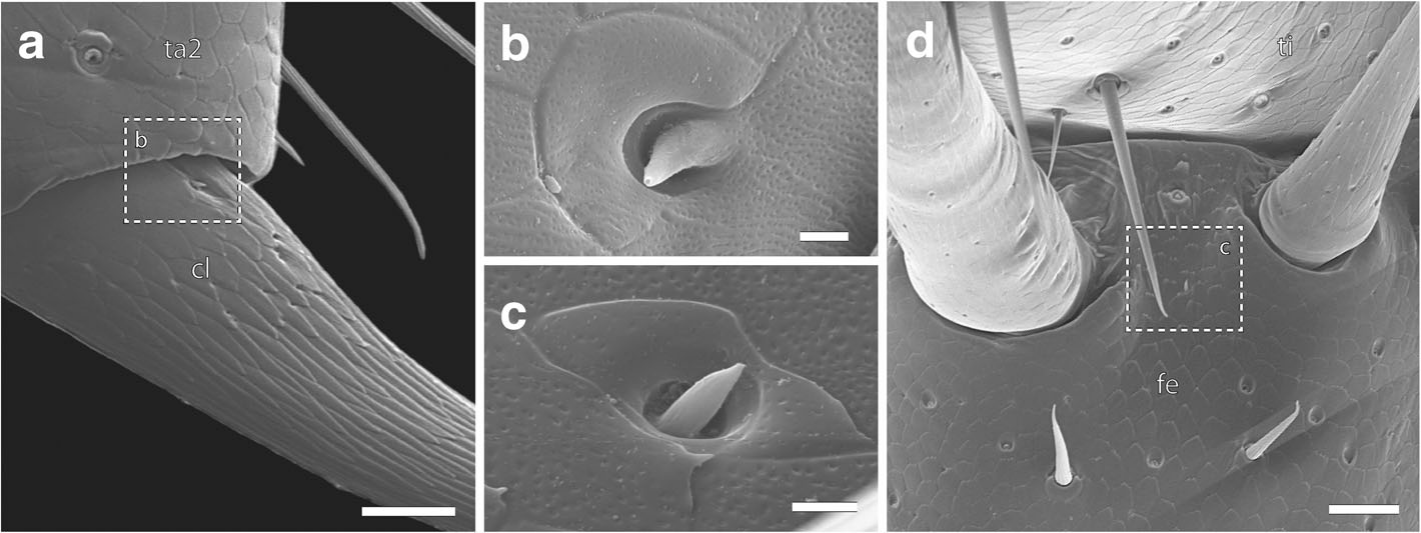
Sensory cones on the ultimate leg. **a** Sensory cone on the ventromedial side of the claw. Note that the claw also features a variety of small pores. Scale bar 25 μm. **b** Detail of proximal portion of the claw as indicated in **a** showing the sensory cone with a terminal pore (arrowhead) located in a small depression close to a crescent-shaped cuticular field. Scale bar 2 μm. **c** Sensory cone on distal femur. Scale bar 2 μm. **d** Ventral femoro-tibial articulation showing the location of a sensory cone on the telopodite. Scale bar 25 μm. Abbreviations: cl claw, fe femur, ta tarsus, ti tibia

Six different types of cuticular sensilla could be identified by SEM analysis (Figs. 5, 6 and 7). In total, there are approximately 300 sensilla present on walking leg 10 (*n* = 3), whereas about 700 sensilla are present on the ultimate leg (*n* = 5). Although the size variation of legs between sexes likely is mirrored in absolute abundance, we found no indications of sexually dimorphic characteristics in terms of sensillar types or relative abundance on either the walking or the ultimate leg. In general, sensilla are distributed over the entire surface of both walking and ultimate legs. On the ultimate leg, however, the medial – telopodal gland associated – face remains mainly devoid of sensilla (Figs. 2d; 3c).

1. Sensillum macrotrichodeum type I (Figs. 2c–e; 3b; 5; 6a, g) is one of the predominant types of sensilla on walking and ultimate legs, constituting nearly a third of the total sensilla count. About 50 (*n* = 2) s. macrotrichodea type I are found on walking leg 10, while the ultimate leg has about 300 (*n* = 3). They possess a crescent-shaped socket and are always associated with one glandular pore adjacent to the socket wall (Figs. 5; 6g). The shaft features a variety of fine and slightly curled ribs, has a mean length of 144 μm (114–173 μm, *n* = 18), and a mean basal diameter of 6.4 μm (4.6–7.9 μm, *n* = 18). The distal tip is gently curved, and no apical pores were detected.

(2). Sensillum macrotrichodeum type II (Figs. 2e; 5; 6h) is only present on walking legs 1–14. About 10–14 sensilla stand oblique-angled in two medial rows along the ventral side of tarsus 2, pointing distally (Fig. 2e). These sensilla possess a finely grooved and slightly contorted shaft of 122 μm in length (112–128 μm, *n* = 4), an average basal diameter of 6.7 μm (6.4–7.1 μm, *n* = 4), and a peculiar crescent shaped, multidentate socket (Figs. 5; 6h), always associated with one glandular pore. No apical pores were detected.

(3). Sensillum mesotrichodeum type I (Figs. 5; 6b) is the most frequent type, constituting more than a third of the total sensilla count. Walking leg 10 bears about 110 these sensilla (*n* = 2), while each ultimate leg bears about 330 sensilla (n = 2). They possess fewer, straight and rather stout ribs (in comparison to s. macrotrichodeum type I) and are on average 44 μm long (37.4–54.7 μm, n = 18) and basally 3.8 μm thick (2.8–3.7 μm, n = 18). Their length becomes gradually shorter on more distal podomeres. No apical pores were detected, but one glandular pore is always present close to the socket (Figs. 5; 6b). Macro- and mesotrichoid sensilla are most abundant on tibia and tarsus 1, and are distributed more or less irregularly on the entire surface of the telopodite with the dorsal and ventral faces showing a row-like aggregation (Fig. 2c–e).

(4). Sensillum mesotrichodeum type II (Figs. 2d; 5; 6c–e) is only present on legs 13–15. These sensilla are on average 34 μm long (25–40.5 μm, n = 4) and have a mean basal diameter of 3.8 μm (3.3–4.0 μm, *n* = 4). The crescent-shaped socket is always associated with two glandular pores (Figs. 5; 6c). Between the pronounced ribs, the shaft displays an intricate, sponge-like surface (Fig. 6c, d). Towards the tip that features a terminal pore (Fig. 6e), the shaft is slightly contorted and occasionally bent. These sensilla, occurring nowhere else, are the only sensilla type present on the medial face of the leg, where they are associated with telopodal glands (Figs. 2d; 3c; 6c). About 4–5 sensilla per podomere are loosely interspersed between the telopodal gland pores, totaling about 20 sensilla on the ultimate leg (*n* = 3, Figs. 2d; 3c).

(5). Sensilla microtrichodea (Figs. 5; 6f) are found on both walking and ultimate legs at the interpodomeric membranes as well as being irregularly distributed on the cuticle, with decreasing abundance towards the claw. Walking leg 10 houses about 40, and the ultimate leg about 50 sensilla (*n* = 2). They have a shallow crescent-shaped socket, few shallow and only slightly curled ribs, and a terminal pore (Fig. 6f). When present, a single glandular pore lies close to the socket. S. microtrichodea are on average 13.9 μm long (13.0–14.5 μm, *n* = 3) and basally 2.8 μm thick (2.6–2.9 μm, *n* = 3).

(6). A single spine-like cuticular cone (sensu *lato* sensory cone) is located in a small depression at the ventromedial base of the claw (Fig. 7a, b). It is present on walking as well as ultimate legs, has an average length of 4.8 μm (4.3–5.3 μm, *n* = 4), and a mean basal diameter of 1.3 μm (1.0–1.5 μm, n = 4). Furthermore, two additional sensory cones are present on the distal margins of the femur and tibia, between the ventromedial spurs of the walking and ultimate leg, adding up to a total of five sensilla per leg (Fig. 7c, d). They have an average length of 4.3 μm (3.1–5.2 μm, n = 3) and an average basal width of 1.4 μm (1.1–1.7 μm, n = 3). At least one cone featured a terminal pore; in other cases, this aspect was inconclusive (Fig. 5).

### Anatomy of the ultimate leg-associated ganglion

The morphology and anatomy of walking leg ganglia of the ventral nerve cord in *Lithobius forficatus* has been covered in detail by Schendel et al. [24]. We therefore will highlight morphological and anatomical characteristics of the ultimate leg-associated ganglion (G15) as well as differences with respect to walking leg ganglion 10 (G10).

**Fig. 8:**
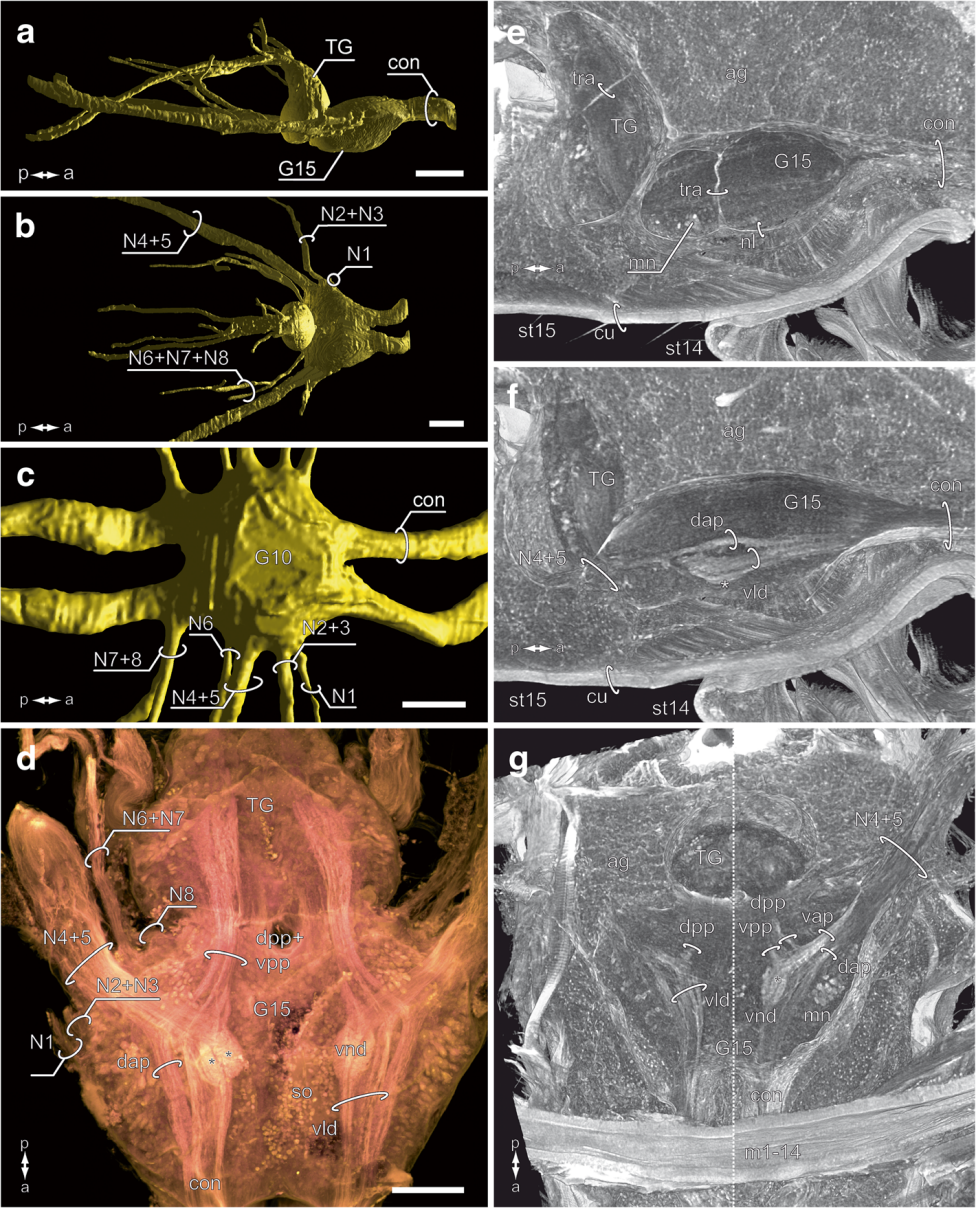
Morphology and anatomy of the ultimate leg-associated ganglion (G15). **a**, **b** 3D reconstruction of G15, the terminal ganglion, and associated nerves. MicroCT analysis, lateral and dorsal views. Note the posteriad directed nerves. Scale bars 200 μm. **c** 3D reconstruction of walking leg-associated ganglion 10, based on microCT analysis, dorsal view (modified after [18]). Note the laterad projecting nerves. Scale bar 200 μm. **d** Autofluorescence preparation of G15 and terminal ganglion. cLSM maximum projection of the ventral part of the ganglion. Within the ganglion, leg afferents from nerves N4 and N5 comprise prominent anteriad projections, including the glomeruli of the ventral neuropilar domain (asterisks) and the ventral lamellate domain (vld). Note that projections proceed into the anterior connectives. In addition, posteriad projections (dpp and vpp) proceed into the terminal ganglion. Scale bar 100 μm. **e–g** Volume visualizations of posterior vnc with G15 and terminal ganglion based on microCT analysis. **e** and **f** sagittal view, **g** horizontal view. The entire posterior vnc is embedded in the tissue of accessory glands (ag) of the male reproductive system. **e** Tracheal ducts (tra) penetrate TG and G15. **f** Leg afferents of N4 + N5 proceed anteriad into the ganglion comprising a dorsal anteriad projection (dap), a ventral lamellate domain (vld), as well as the ventral neuropilar domain (asterisk). **g** Different virtual, horizontal sections showing afferents from N4 + N5 splitting into a dorsal and ventral anteriad projection (dap and vap, right half), the ventral lamellate domain (vld, left half), and the ventral neuropilar domain (vnd, right half). Dorsal and ventral posteriad projections (dpp and vpp) target the terminal ganglion. Note the highly contrasted somata of motoneurons (mn, right half). Abbreviations: ag accessory glands, con connective, cu cuticle, dap dorsal anteriad projection, dpp dorsal posteriad projection, G ganglion of the vnc, m1–14 transversal muscle 1 of trunk segment 14, mn motoneurons, N nerve, nl neurilemma, so somata, st sternite, TG terminal ganglion, tra tracheal ducts, vap ventral anteriad projection, vld ventral lamellate domain, vnd ventral neuropilar domain, vpp ventral posteriad projection

In comparison with regular walking leg ganglia, G15 is stouter and caudally bounded by the terminal ganglion (Fig. 8a, b). The most obvious morphological difference is the arrangement and position of nerves. Whereas in walking leg ganglia the nerves protrude laterally (Fig. 8c), they are directed distinctly posteriad to posteriolaterad in G15, flanking the terminal ganglion (Fig. 8a, b, f). Seven to eight discernible nerves are associated with each hemiganglion (Fig. 8b, f): three nerves (N1–N3) are proximally fused or lie closely together, two thick nerves (N4 + N5) are fused to a prominent nerve bundle almost as thick as a connective, and two nerves (of uncertain composition) emanate from the posteriormost margin of the ganglion, close to the terminal ganglion (in walking leg ganglia the situation is more obvious, compare Fig. 8c). In most of our preparations we detected only one joint nerve root posterior to N4 + N5; only in a single specimen was a vestigial nerve observed (Fig. 8f). Thus, N8 is either reduced or fused to the joint nerve root of N6 + N7 (Fig. 8b). The entire ganglion is surrounded by a thick neurilemma (Fig. 8e). Both hemiganglia are entirely fused medially. The midline of the ganglion, however, features a number of dorsoventral penetrations sheathed by neurilemma (Fig. 8e), probably representing tracheal ducts. In general, the anatomy of the ultimate leg ganglion is characterized by interweaving neurites without distinct anterior and/or posterior commissures, denser neuropilar regions in the ventral part of the ganglion, and a ventrally thicker cortex of somata (Fig. 8d–g). The connectives associated with the adjacent ganglion in front (G14) are highly structured by longitudinal neurites, but essentially absent from the terminal ganglion behind (Fig. 8d–g). The transition is only discernible due to a necking of the ganglion (Fig. 8a, b, d–g).

**Fig. 9:**
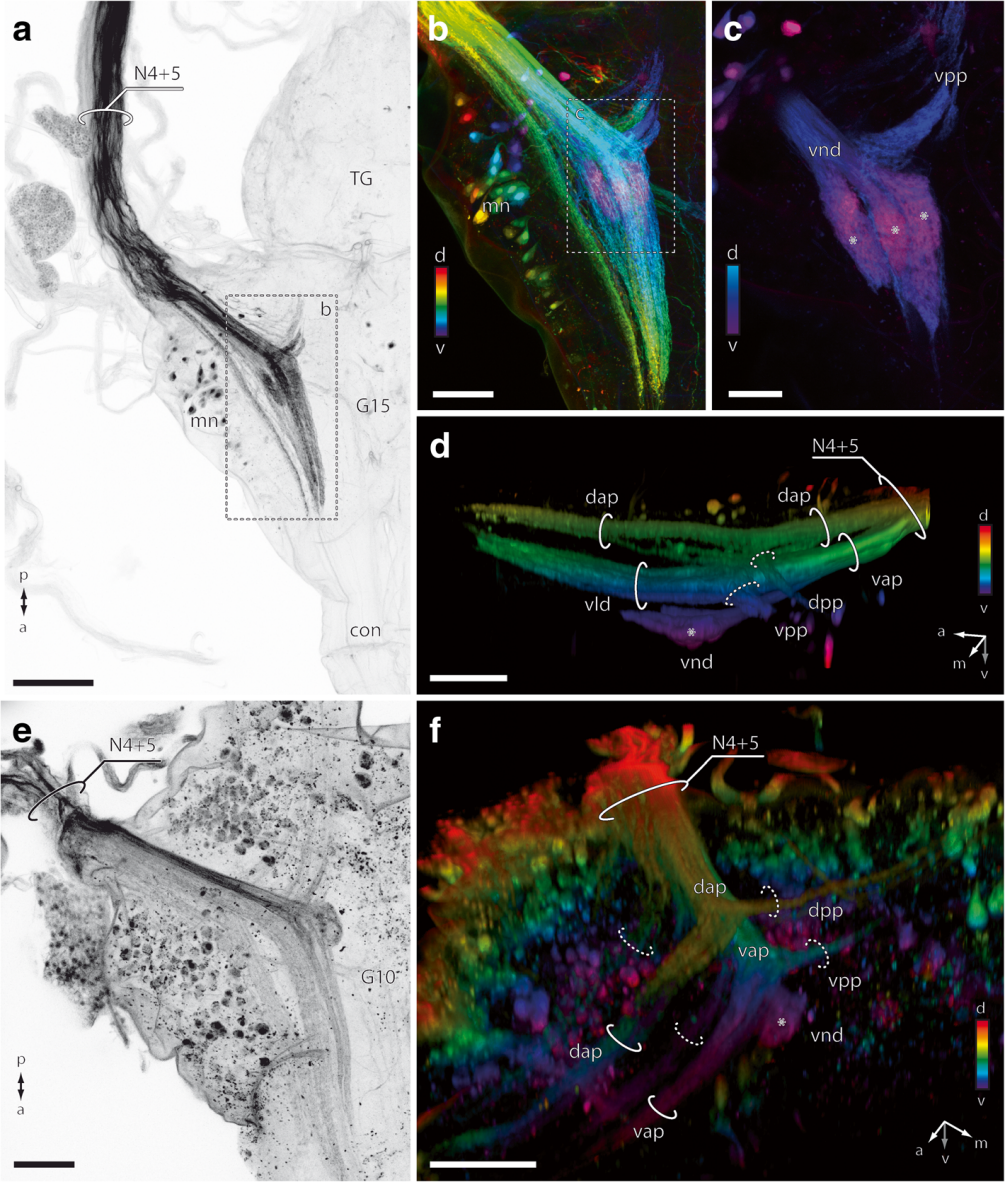
Backfill experiments. **a**–**d** Backfill of the ultimate leg nerves N4 + N5. **e**, **f** Backfill of the walking leg 10 nerves N4 + N5. **a** Black/white inverted cLSM maximum projection of ultimate leg nerve backfill, view from dorsal, right hemiganglion. Note the intensely labeled motoneurons (mn) in the lateral cortex of G15. Scale bar 100 μm. **b** Backfill of ultimate leg nerve **a** depth color coded. The ventralmost area (purple) is characterized by the vnd (compare **c**). Scale bar 50 μm. **c** Subsection of **b**, the ventral neuropilar domain features three distinct glomerular subunits (asterisks). Scale bar 25 μm. **d** Depth color-coded 3D visualization (Amira) showing the dorso-ventral organization of N4 + N5 afferents, view from medial. N4 + N5 split into a dorsal anteriad projection (dap) and a ventral anteriad projection (vap). Both projections each branch off a small posteriad projection (dorsal and ventral posteriad projections, dpp and vpp). The vap splits into the ventral lamellate domain (vld) and the ventral neuropilar domain (vnd, asterisk). Scale bar 50 μm. **e** Black/white inverted cLSM maximum projection of waking leg nerve backfill, right hemiganglion. Note the intensely labeled neurons in the lateral and ventral cortex of G10. Scale bar 50 μm. **f** Depth color coded 3D visualization of backfill (**e**) showing the dorsoventral organization of N4 + N5 afferents, view from dorsomedial. The general branching pattern is similar to the ultimate leg-associated ganglion (compare **d**). However, the ventral anteriad projection is not differentiated into a lamellate domain, only giving rise to the ventral neuropilar domain (vnd, asterisk) which is far smaller in comparison. Scale bar 50 μm. Abbreviations: con connective, cu cuticle, dap dorsal anteriad projection, dpp dorsal posteriad projection, G ganglion of the vnc, mn motoneurons, N nerve, TG terminal ganglion, vap ventral anteriad projection, vld ventral lamellate domain, vnd ventral neuropilar domain, vpp ventral posteriad projection

Specific projection areas of the ultimate leg nerves are detectable by autofluorescence (Fig. 8d), microCT analysis (Fig. 8e–g), and backfill experiments (Fig. 9). From the joint nerve root of N4 + N5, two major projections traverse the ganglion in anteromediad direction: the dorsal and the ventral anteriad projection (dap and vap; Figs. 8e–g; 9a–d). Close to the nerve root, both projections give rise to a smaller branch (dorsal and ventral posteriad projection), which take an almost 90° turn and target the neuropil of the terminal ganglion (dpp and vpp; Figs. 8d, g; 9a–d). The majority of neurites, however, proceed anteriad and target different areas of the neuropil (Figs. 8d–g; 9a–d). The dorsal anteriad projection (dap), located along the paramedian plane of the ganglion, is relatively thin and proceeds anteriad without further arborizations (Figs. 8d–g; 9d). The ventral anteriad projection (vap) is considerably thicker and splits up into two separate domains: the ventral lamellate domain (vld) that comprises at least three parallel longitudinal lamellae projecting anteriad (Figs. 8d–g; 9d), and the ventral neuropilar domain (vnd) that comprises at least three glomerular neuropils (Figs. 8d, f, g; 9c, d). The vnd is located in the ventralmost part and close to the soma cortex sheathing the ventral surface of the ganglion (Figs. 8d asterisk; 9c, d). Although labeled neurites could only be traced within the respective ganglion, parts of dap and vap likely proceed along the ventral nerve cord to at least the adjacent ganglion in front (Fig. 8d, f, g).

The ganglion associated with walking leg 10 (G10) is structured in a generally similar way in terms of innervations and projections (Fig. 9e, f). However, in the walking leg both anteriad projections are less pronounced and the dap does not appear lamellate. Also, the vnd is distinctly smaller and not differentiated into glomerular subunits, thus featuring only a single domain (Fig. 9f). Moreover, the volume of the vnd in G10 accounts for only about a fifth of the ultimate leg vnd (compare Fig. 9d vs. f).

## Discussion

As outlined by Kenning et al. [1], centipede ultimate legs are special, and *Lithobius forficatus* is no exception. Several, not mutually exclusive pathways of morphological modifications and behavioral adaptations can be distinguished in centipedes. Many species possess elongated, even multi-annulated ultimate legs (Scutigeromorpha, *Newportia* spp.), some species possess thickened or pincer-like ultimate legs (e.g. Scolopendromorpha), and in many species sexual dimorphisms occur (in particular Geophilomorpha). The ultimate legs of *L. forficatus* contrast with other, more drastic examples of ultimate leg transformations in centipedes [1] as they in many aspects still resemble a regular walking leg. In general, in *L. forficatus* there is a continuous increase in leg length, in particular in legs 14 and 15 (Fig. 1a) [2]. Thus, given the presence of telopodal pores and coxal organs, the three most posterior pairs of walking legs (12–14) as well as the ultimate legs (15) may pose as an example of a gradual morphological modification in contrast to regular walking legs. In comparison to the walking leg 10, the ultimate leg features a series of distinctions such as the leg musculature, the glandular organization, sensillar configuration, and the associated nervous system as discussed in the following.

### External morphology, musculature, and tendons

In addition to size, one of the major differences in podomere configuration between walking and ultimate legs is the division of the tarsi. According to Zapparoli and Edgecombe [4], in the genus *Lithobius* legs 1–13 exhibit a clearly visible tarsal articulation between the tarsomeres, although this articulation is not complete (i.e. the joint is complete around all but a small extent dorsally). This is in concordance with comprehensive anatomical descriptions of *Lithobius forficatus* by Rilling [23, 34], as well as descriptions of Lithobiomorpha by Eason [2], Lithobiidae by Barber [3], and this contribution. Only the tarsi of leg pairs 14 and 15 show a complete separation and thus constitute distinct podomeres, providing new degrees of freedom in leg posture and movement. The functional implications are diverse but conjectural at this time, particularly as most of the leg joints lack fixed rotational axes which allows for a variety of displacements (compare [34]). However, it seems reasonable to assume that the enhanced maneuverability and flexibility achieved by the fragmentation of the tarsi is significant in light of the defensive performance of the ultimate legs. Which role the presumptive second tendon of the tibio-tarsal articulation plays however, remains uncertain, but likely it supports elevation of the tarsi.

Another prominent difference in podomere configuration is found in the coxa. In the walking leg, the coxa forms an incomplete ring, which allows the trochanter to be pulled in the soft pleural flanks as part of the remotor movement [35]. In the ultimate leg, the entire coxa is turned caudally, leaving the lateral (formerly anterior) side as a rigid cuticular ring. Moreover, while several pro- and remotor muscles are reduced (e.g. transversal muscle m1 as well as sterno-trochanteral muscles m37), levators and depressors (i.e. coxo-trochanteral muscles m40 and m41) are substantially larger [34]. This effectively allows only for up and downward movements, but less so for lateral motion. Although ultimate legs do not participate in locomotion in terms of propulsion, they play a pivotal role in the process by stabilizing the body. Ultimate legs are always stretched backwards. When a centipede turns to one side, the ultimate legs swing to the other side resulting in a faster turn. Experiments with animals that have only a single ultimate leg left give further support to this hypothesis: the remaining ultimate leg is always held along the median plane of the body in order to keep the centre of mass and hence balance [8].

### Coxal organs

All centipedes but Scutigeromorpha feature coxal organs – specialized epithelia and pores that are in most cases associated with the ultimate leg coxae (e.g. [4, 11, 36–40]; apomorphic character for Pleurostigmophora [41]). Remarkably, in most species of Lithobiomorpha the coxal organs are not restricted to the last pair of legs, but are present on the coxae of leg pairs 12–15 (secondarily modified in some species to the last two, three or five leg pairs [41]). According to Herbst [42], coxal organs serve a defensive function, facilitating the autotomy of legs, while Verhoeff [8] attributed them a function in supporting leg regeneration, or even in prey capture. Their relevance still is not conclusively resolved. Littlewood [43, 44] interpreted lithobiomorph coxal organs to be release sites of pheromones. However, the presence of transport epithelia, as well as ecological data and behavioral observations suggest a role in atmospheric water uptake (as proposed by Rosenberg and Bajorat [45]). In fact, species living in rather dry habitats (*Hessebius* spp.) feature a low number and diameter of coxal pores, while very large diameters were reported in species living near to streams [46]. In any case, the biological importance of coxal organs is demonstrated by being present on the last four pairs of legs.

### Telopodal organs

Telopodal organs were originally interpreted as pheromone glands, because pores and associated glandular systems are thought to be absent in the anamorphic larval stages [12, 13, 47]. However, Lewis and Yeung [48] documented the changes in the distribution of telopodal pores during the development of *L. microps* and have shown that during the larval and postlarval stages, each last pair of legs houses a certain number of telopodal pores. In successive stadia and the anamorphic addition of leg pairs, the number of telepodal pores increases on the respective last pair of legs (e.g. leg 12 in stage 4, with legs 13–15 only present as limb buds [49, 50]), but also decreases on the former ultimate legs.

Also termed defense glands, these closely aggregated glandular organs become effective in predation avoidance by the secretion of a sticky, slowly hardening substance, trapping any opponent [5, 8, 11]. Besides its adhesive quality, little is known about the biochemical composition or physical properties of the secretion. Based on histochemical staining experiments, Blower [12] described the secretion of the telopodal glands as amber-colored substance that hardens rapidly and forms fibers after being discharged. Staining reactions revealed that the telopodal secretion contains lipoids or proteins, or a mixture of both. According to Verhoeff [8], a single podomere of *Lithobius mutabilis* accommodates about 200 glandular pores which total up to approximately 800–1200 pores per ultimate leg. Kästner [51] stated that *L. forficatus* has over 500 pores per ultimate leg. Given the 4000 pores in *L. forficatus* as shown in this contribution, we believe that it is possible, if not probable, that the specimens Verhoeff and Kästner analysed were still in their late anamorphic, or early epimorphic stages.

### Sensilla typology and distribution

Epidermal sensilla are widespread on the cuticle of centipedes, and are mostly aggregated on the antennae, mouthparts, legs, and gonopods [52]. However, only few sensilla types are comprehensively understood with regard to their ultrastructure and function (summarized by [52–54]). The external morphology of antennal sensilla has been investigated for at least one species in each of the higher-taxonomic groups of centipedes: *Scutigera coleoptrata* [55], *Lithobius forficatus* [5, 21], *Craterostigmus tasmanianus* [56], *Cryptops hortensis* [57], *Scolopendra oraniensis* [58], and *Geophilus flavus* [54, 59–62]. The most comprehensive accounts on *L. forficatus* [5, 21] revealed approx. 2400 sensilla on each antenna. About 2000 trichoid sensilla are present, each with a length of 100–150 μm and featuring a terminal pore. The angle to the antennal cuticle is more or less 90°. The shaft bears numerous spiral ribs and it is basally encompassed by a crescent-shaped socket, and two small glandular pores. Sensory cells possess short dendritic outer segments exhibiting tubular bodies (mechanoreception) and long dendritic outer segments that project towards the terminal pore (chemoreception) [21]. In addition, about 80 sensilla basiconica, 80 s. brachyconica, and 240 s. microtrichodea are present on each antenna.

Primarily, trichoid sensilla were found on walking and ultimate legs. We classified subtypes based on differences in length of their shafts (macro-, meso-, and microtrichoid). The subtype s. macrotrichodeum II with a multidentate socket was exclusively found on walking legs. Apart from the dentate socket, s. macrotrichodea II are slightly smaller than the dominant s. macrotrichodea I (present on all legs). As we detected no terminal pores, an exclusive mechanoreceptive function is presumed. Type I s. mesotrichodea are present on all legs and are assumed to function exclusively as mechanoreceptors. Type II s. mesotrichodea II are only present on legs 13–15 and are the only sensilla on the legs which are associated with two glandular pores, a feature that is usually found on antennal s. trichodea [5]. The only similarity between s. mesotrichodea I and II is their shaft length. Other features like the presence of a terminal pore, the association with two glandular pores, as well as the exclusive distribution clearly differentiates these subtypes. Similar sensilla were not described on the antennae of *L. forficatus* [5]. The presence of a terminal pore clearly indicates a chemosensory function. The sponge-like, serrated appearance of the shaft between the ribs might suggest an additional olfactory function. Interestingly, this morphology is similar to the beak-like sensilla in *Scutigera coleoptrata* [55, 63] that are thought to serve an olfactory function in scutigeromorph centipedes. Whether these cuticular specializations are associated with pores or a specialized cuticle remains unclear without ultrastructural investigation. The location of s. mesotrichodea II interspersed between the pores of telopodal glands also indicates that these sensilla might function as a quality assessment of telopodal gland secretion. As no sexual dimorphic characteristics were found, a role in chemical recognition/communication is unlikely, but cannot be ruled out. Sensilla microtrichodea are present on all legs and the antennae [5], with minor differences in shaft length. Antennal s. microtrichodea are present in all investigated pleurostigmophoran centipedes arranged at the base of antennomeres, and are thought to function as proprioceptors providing information on the position of the antenna [5, 56, 57, 62]. Only in *L. forficatus* were antennal s. microtrichodea described without a terminal pore and lacking long dendritic outer segments [5]. However, on walking and ultimate legs, we detected terminal pores, thus an additional chemoreceptive function can be assumed.

Apart from trichoid sensilla, small sensory cones were found on all legs, on the claw, as well as the distal femur and tibia. The very short shaft, the slightly sunken appearance, and the presence of a terminal pore are (at least) comparable to sensory cones in Scolopendromorpha [20, 58]. The term sensory cone was introduced to describe a very small, typologically incomprehensive class of antennal sensilla [55]. Therefore, it is unwarranted to refer to them as homologous sensilla. The presence of similar cones on the claw was also detected in *Lithobius obscurus* and *Craterostigmus tasmanianus* (termed ‘basiconic sensilla’ [64]), and a homology between those sensilla in Lithobiidae and either the subsidiary spine or posteroventral spine of Henicopidae was proposed [64]. However, based on size, shaft surface and insertion, we reject this terminology to avoid unjustified homology hypotheses (compare [52]). While they may function as contact-chemoreceptive sensilla on the claw given their location and presence of a terminal pore, they will hardly come into contact with any substrate on the telopodite. Their function is thus uncertain, but may be thermo- or hygroreceptive in nature, given their external-morphological similarity to thermoreceptors in hexapods [65, 66]. Bauer [17] showed that in *L. forficatus* hygroreception is mainly located on the tarsi, while thermoreception is mostly mediated by basal antennomeres [16, 34]. Again, without complementary ultrastructural investigations, any functional conclusions are a matter of speculation.

### Neuroanatomy of the ultimate leg-associated ganglion

As demonstrated by Schendel et al. [24], walking leg ganglia 1–14 of *L. forficatus* are of rather uniform morphology and architecture which is in accordance with previous investigations and descriptions [34, 67–70]. The ganglion associated with the ultimate legs (G15), however, shows a variety of anatomical peculiarities.

Only in the ganglion of the ultimate leg, leg nerves are directed posteriad, which corresponds to the transformation of the coxa and posture of the respective telopodites. As in the walking leg ganglia, G15 houses several large projections and termination sites associated with leg nerves N4 + N5. However, the ventral neuropilar domain (vnd) of G15 has a multiglomerular organization as opposed to a uniglomerular organization of G10 (compare Fig. 9e, f, and [24]). Moreover, the volume of the G15 vnd is at least five times as large as the counterpart in the walking leg-associated G10. Another substantial architectural difference lies within the ventral anteriad projection (vap). While in G10 the vap remains as an inconspicuous bundle of longitudinal fibers giving rise to the vnd, the vap of G15 is condensed to a series of discrete longitudinal, parallel lamellae, hence termed the ventral lamellate domain (vld). In general, tetraconate (Crustacea and Hexapoda) vnc ganglia associated with appendages possess particularly organized neuropilar domains. For example, the thoracic ganglia of Hexapoda feature sensory neurites and associated neuropils in the ventral part of the ganglion, the so-called ventral association center (vac) [25, 71–73]. This organization and corresponding tract patterns are also found in crustaceans (e.g. [74–76]). Within the vac, different sensory modalities terminate in different regions, e.g. afferents from mechanoreceptive sensilla project to distinct regions of the neuropil and given the specific sensory modality, an ordered structure of neuropilar areas is usually observed along a gradient [25, 26, 77]. While in locusts and crayfish the vac is either fused or strongly interconnected by commissural fibers, each hemiganglion of *L. forficatus* features distinct neuropilar regions without obvious contralateral connections.

Intriguingly, the neuropilar domain in the G15 of *L. forficatus* is organized into two domains (ventral neuropilar and lamellate domains). The principle of a bipartite architecture in processing afferents is mostly known for and well documented in the mandibulate deutocerebrum. Innervated by a variety of antennal sensilla of different modalities, it is typically composed of a glomerular neuropil processing chemosensory information and (at least one) mechanosensory neuropil [22, 25, 27, 28, 31, 32], which is also the case in the pectines-associated neuromere of scorpions [78, 79]. In terms of sensory neurobiology, glomeruli are (with few exceptions) usually associated with the processing of chemoreceptive stimuli in a chemotopic manner (e.g. [25, 28, 29, 32]), whereas a highly ordered rectilinear, striated and multilayered arrangement of fibers with perpendicular arborizations can be attributed to the processing of mechanosensory information in a somatotopic manner [25, 80–82]. The bipartite organization of the deutocerebrum of *Lithobius forficatus* features the deutocerebral chemosensory lobes (typically composed of 43 glomeruli) and the corpus lamellosum (mechanosensory neuropil) that is composed of at least four neuropilar lamellae [19, 22, 67]. Although numbers are much lower, the organization and the specific architecture of the ventral neuropilar and lamellate domains show a strong resemblance to the deutocerebral neuropils. Taking the characteristics and elaboration of primary processing centers associated with antenna, walking leg and the ultimate leg into account, it is evident that as a general consequence, a pronounced input from cuticular sensilla shapes primary processing neuropils. The increase in total volume and in volume of the associated neuronal substrate in G15, the increase in glomerular number, and the differentiation into lamellae thus indicates an enhanced sensory performance that is mirrored by the higher abundance of cuticular sensilla. Given the incremental enlargement of legs 12–14 as well as the augmentation with sensilla and epidermal glandular pores, a likewise gradual increase in sensory performance as well as volume of associated processing centers in ventral nerve cord ganglia is to be expected, but requires further examination.

## Conclusion

One of the most prominent differences between walking and ultimate legs, one that emphasizes their disparity and renders them a promising model for sensory biology, is the sensilla, which are distinct both in terms of quantity and quality, as well as their underlying neuronal substrate. Although not quite twice as long, the ultimate leg houses nearly three times as many sensilla as the walking leg. In comparison, the antennae of *L. forficatus* are again roughly twice as long as the ultimate legs, and house three times more sensilla (~ 2400 [5]). Yet, not only the abundance, but also the density of sensilla is much higher on the ultimate leg, even though the medial telopodal gland-associated faces are free of all but one sensilla type. Thus, a functional correlation of s. mesotrichodea II with the telopodal gland’s secretion is reasonable. Based on the higher abundance of sensilla, ultimate legs can be expected to demonstrate a higher mechano- and chemoreceptive performance.

While aspects of the external morphology may undergo transformations more readily, structure and organization of the underlying neuronal substrate are far more conservative, especially in terms of evolution [83]. Modifications are thus of particular importance and interest, such as the emergence of elaborate and compartmentalized domains (e.g. glomeruli) in neuromeres other than the deutocerebrum in the same species. All appendages and associated ganglia of the nervous system are serially homologous. The question remains whether morphological coherence of the primary processing neuropils in the deutocerebrum versus the ultimate leg-associated ganglion can be regarded as a corollary of serial homology or rather reflects a convergence expressed in the operation of constructional constraints. Given a certain near-threshold number of syntonic sensory neurons whose axons converge on a distinct chemosensory domain, a “spherical” neuropil is the most space-efficient option. Yet, what makes the situation in *L. forficatus* remarkable is the entire sensory framework of glomerular and lamellate neuropils, and its specific correspondence to the deutocerebral organization.
